# Extremely Sensitive Microwave Sensor for Evaluation of Dielectric Characteristics of Low-Permittivity Materials

**DOI:** 10.3390/s20071916

**Published:** 2020-03-30

**Authors:** Tanveerul Haq, Cunjun Ruan, Xingyun Zhang, Shahid Ullah, Ayesha Kosar Fahad, Wenlong He

**Affiliations:** 1 School of Electronic and Information Engineering, Beihang University, Beijing 100191, China; tanveerulhaq@buaa.edu.cn (T.H.); luckyzhang@buaa.edu.cn (X.Z.); shahidkhan@buaa.edu.cn (S.U.); ayeshakosar@buaa.edu.cn (A.K.F.); 2 Beijing Key Laboratory for Microwave Sensing and Security Applications, Beihang University, Beijing 100191, China; 3 College of Electronics and Information Engineering, Shenzhen University, Guangdong 518060, China; wenlong.he@szu.edu.cn

**Keywords:** Microwave sensor, CSSSR, permittivity, permeability, material under test, transcendental equation

## Abstract

In this paper, an extremely sensitive microwave sensor is designed based on a complementary symmetric S shaped resonator (CSSSR) to evaluate dielectric characteristics of low-permittivity material. CSSSR is an artificial structure with strong and enhanced electromagnetic fields, which provides high sensitivity and a new degree of freedom in sensing. Electromagnetic simulation elucidates the effect of real relative permittivity, real relative permeability, dielectric and magnetic loss tangents of the material under test (MUT) on the resonance frequency and notch depth of the sensor. Experiments are performed at room temperature using low-permittivity materials to verify the concept. The proposed design provides differential sensitivity between 102% to 95% as the relative permittivity of MUT varies from 2.1 to 3. The percentage error between simulated and measured results is less than 0.5%. The transcendental equation has been established by measuring the change in the resonance frequency of the fabricated sensor due to interaction with the MUT.

## 1. Introduction

Microwave sensors have quick response time, wide sensing range, high accuracy, no effect of temperature, and are suitable for any climate, therefore these sensors are widely used in different industries like agriculture [1,2], the biomedical sector [3,4,5,6], and electronics [7,8,9,10]. Recently microwave sensors based on metamaterial (MTM) and complementary MTM structures have achieved high level of sophistication due to the novel properties of MTM such as negative effective permittivity [11], negative effective permeability [12], negative index of refraction [13,14] and backward wave propagation [15]. Initially, these novel properties were achieved using a split-ring resonator (SRR) [16] and complementary split-ring resonator (CSRR) [17]. Later, these artificial structures were used to design microwave sensors for testing liquids [18,19,20,21,22], measurement of thickness [23,24,25,26], relative humidity [27], displacement [28,29,30,31,32], rotation [33,34,35,36], strain [37,38,39], permittivity and permeability [40,41,42]. The main advantages of SRR- and CSRR-based sensors are the small size with high sensitivity, lower cost with robustness, and high precision. The performance of the planar microwave sensor depends on the resonating structure as well as the hosting transmission line [43,44,45,46,47,48]. 

In [43], an SRR with a resonance frequency of 0.87 GHz is used to design a differential sensor with a size of 0.1λ_g_^2^ for liquid characterization and an average sensitivity of 0.91% is achieved. In [44] a stepped impedance resonator (SIR) loaded with a microstrip transmission line is used to design a differential sensor with a size of 0.046 λ_g_^2^ for comparison of three samples simultaneously, and an average sensitivity of 1.81% is achieved. In [45], a CSRR with resonance frequency of 1.7 GHz is utilized to design a differential sensor with a size of 0.098 λ_g_^2^ for dielectric characterization and the average sensitivity of 1.96% is achieved. In [46], an SRR loaded with a microstrip transmission line is used to design a differential sensor with a size of 0.034 λ_g_^2^ for comparison of dielectric samples and an average sensitivity of 3.4% is achieved. In [47], an SIR with resonance frequency 6.1 GHz is used to design a differential sensor with a size of 0.15 λ_g_^2^ for dielectric characterization and the average sensitivity of 8.8% is achieved. In [48], a dual notch microwave sensor is designed based on complementary bisymmetric SRR with a size of 0.05 λ_g_^2^ for evaluation of dielectric substrates and an average sensitivity of 29.9% is achieved. The aforementioned sensors are based on permittivity perturbation of MUT and have sensitivity limitations due to small resonance frequency. Two parameters are important for sensitivity comparison, the first being the shift in resonance frequency of the sensor due to interaction with MUT with respect to the free space resonance frequency, and second being the relative permittivity of MUT. The sensitivity of various state of art sensors [49,50,51,52,53,54] based on the resonance frequency shift is tabulated in Table 1.

If two sensors are loaded with identical MUTs then the sensor with high resonance frequency will give higher shift in resonance frequency. Therefore, we have designed a sensor based on a complementary symmetric S-shaped resonator (CSSSR), which provides free space resonance frequency of 15.17 GHz. The CSSSR is a negative image of an S-shaped resonator, which has already been used as an end reflector [55], bandpass filter [56], dual-band filter [57], waveguide filter [58], tunable metamaterial [59], and angular velocity sensor [60]. In this work, the CSSSR is coupled with a microstrip transmission line to design a sensor to test the low permittivity materials. The change in resonance frequency and notch depth of the sensor is calculated numerically by permittivity perturbation, permeability perturbation, dielectric loss and magnetic loss perturbation in Section 2. After fabrication, the sensor is tested for sensing low permittivity dielectric material using a vector network analyzer (VNA) and the measured results are formulated in Section 3 and concluded in Section 4.

## 2. Design and Simulation of Proposed Sensor

**Figure 1:**
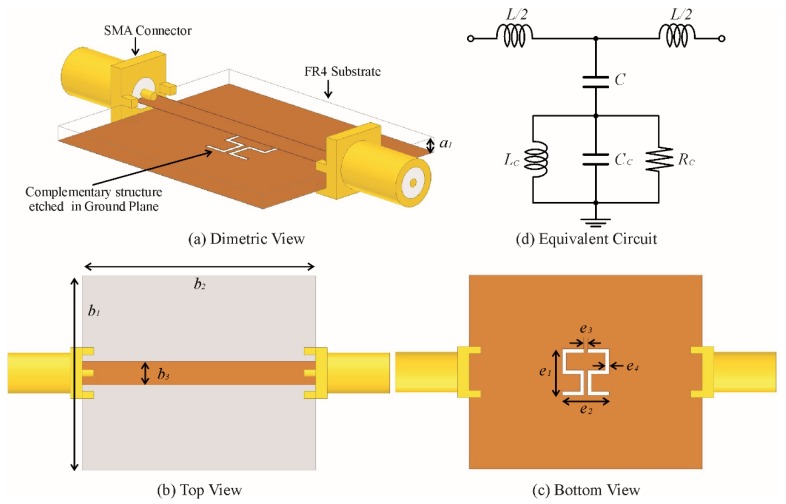
(**a**) Sensor design based on FR4 epoxy substrate (*a_1_* = 1.6 mm). (**b**) Top view of sensor (*b_1_* = 25 mm, *b_2_* = 30 mm, *b_3_* = 3 mm). (**c**) Bottom view of sensor (*e_1_* = e_2_ = 6 mm, *e_3_* = e_4_ = 0.5 mm). (**d**) Equivalent circuit model with L and C for the unit length inductance and capacitance of the microstrip transmission line respectively and (RLC)_C_ for the complementary symmetric S-shaped resonator (CSSSR).

The design of the proposed sensor is based on a microstrip transmission line which is directly coupled to the CSSSR as shown in Figure 1a. For proper excitation of resonating structure, the microstrip transmission line (3 mm) and the CSSSR are printed on the top and bottom layer of the substrate as shown in Figure 1b,c respectively. The CSSSR structure is equivalent to parallel combination of resistance (*Rc*), inductance (*Lc*) and capacitance (*Cc*). The microstrip transmission line is equivalent to the inductance (*L*) and the thickness of the substrate provides the capacitance (*C*) between the transmission line and the CSSSR. The equivalent lumped element circuit model of the coupled structure is shown in Figure 1d, the resonance frequency of the equivalent circuit can be calculated using the following relation [61]:

(1) $$f_{r}=\frac{1}{2\pi \sqrt{L_{c}\left(C+C_{c}\right)}}$$

**Figure 2:**
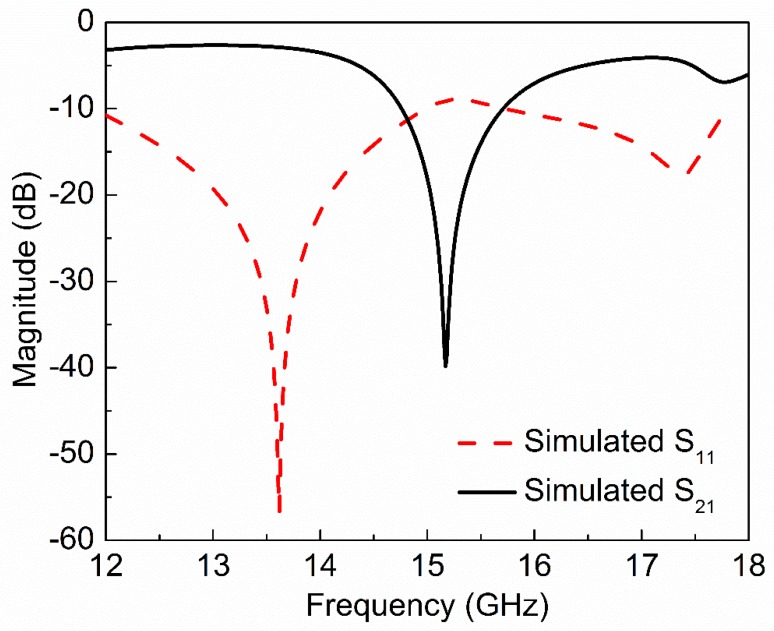
Simulated reflection (S_11_) and transmission (S_21_) coefficient of the proposed sensor based on CSSSR. The proposed sensor shows resonance at 15.17 GHz with notch depth −39.84 dB.

The 3D model of the proposed sensor is simulated in ANSYS HFSS software with the simulation conditions given in ref. [58]. The proposed sensor shows a resonance at 15.17 GHz with notch depth of –39.84 dB as shown in Figure 2. The unloaded quality factor of the proposed sensor is 505 which can be calculated by the following relation [62]:

(2) $$Q=\frac{f_{r}}{∆f_{3dB}}$$

The resonance frequency of the proposed sensor can be shifted to lower frequencies due to interaction with the material under test (MUT) which is the basic principle of microwave sensors. At the resonance frequency of the CSSSR, the stored electric field (*E_0_*) and magnetic field (*H_0_*) are equal to each other. When a MUT interacts with the CSSSR, it disrupts the equilibrium of the stored electromagnetic fields and generates the new electric field (*E_1_*) and magnetic field (*H_1_*) which causes it to change the resonance frequency. This change in resonance frequency (∆*f_r_*) depends on the change in permittivity (∆ɛ), permeability (∆µ) and volume (ʋ) of the MUT, which can be expressed mathematically as given in reference [63]:

(3) $$\frac{∆f_{r}}{f_{r}}=\frac{\int_{\upsilon }(∆\varepsilon E_{1}.E_{0}+∆\mu H_{1}.H_{0})d\upsilon }{\int_{\upsilon }(\varepsilon_{0}{\left|E_{0}\right|}^{2}+\mu_{0}{\left|H_{0}\right|}^{2})d\upsilon }$$

**Figure 3:**
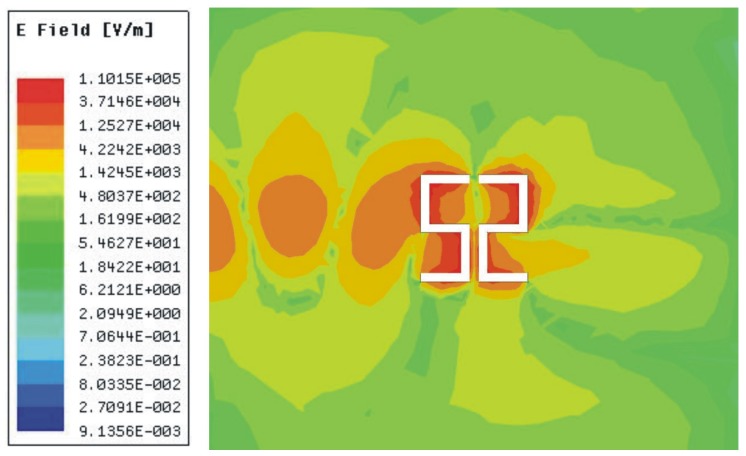
Magnitude of electric field at the resonance frequency of the proposed sensor. The electric field is concentrated at the edges of the CSSSR and its maximum value is 1.10 x 10^5^ V/m.

**Figure 4:**
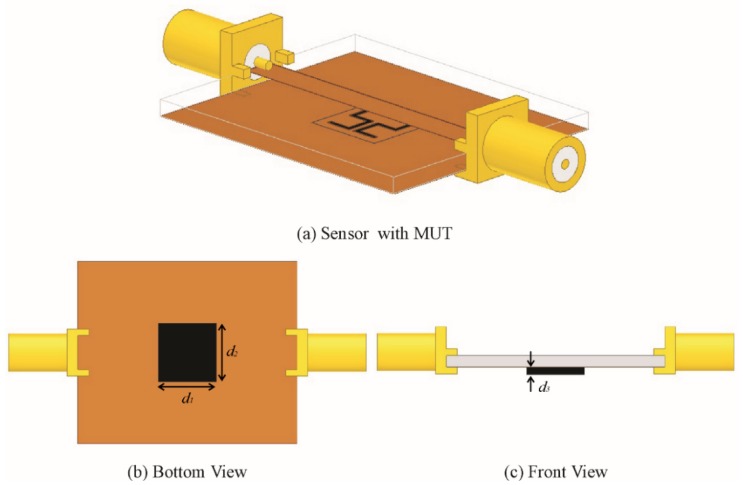
(**a**) Sensor based on CSSSR with material under test (MUT) in the ground plane with air gap (35 µm). (**b**) Bottom view of the sensor with MUT (*d*_1_= 10 mm, and *d*_2_ = 10 mm). (**c**) Front view of the sensor with MUT (*d*_3_ = 1 mm).

**Figure 5:**
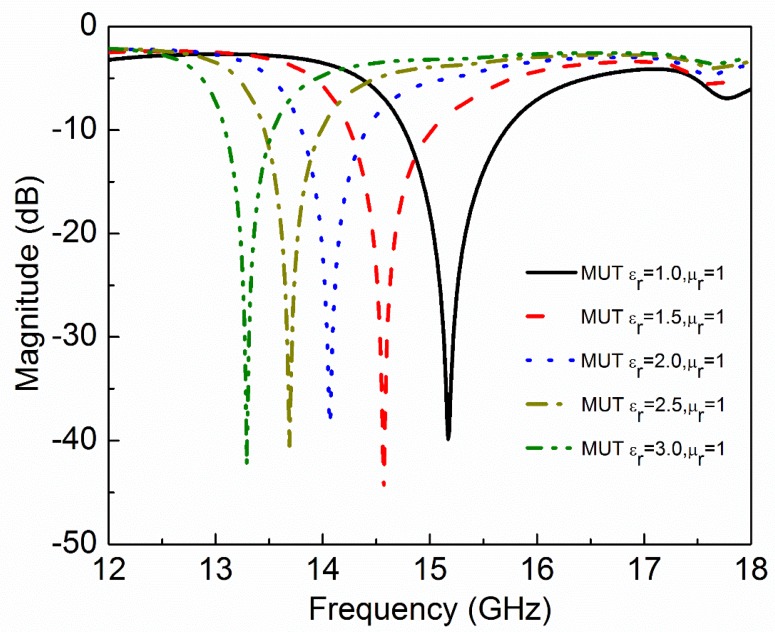
Simulated transmission coefficient (S_21_) of the sensor due to interaction with the MUT with different values of permittivity (ε_r_). As the relative permittivity of MUT increases, the resonance frequency decreases.

**Figure 6:**
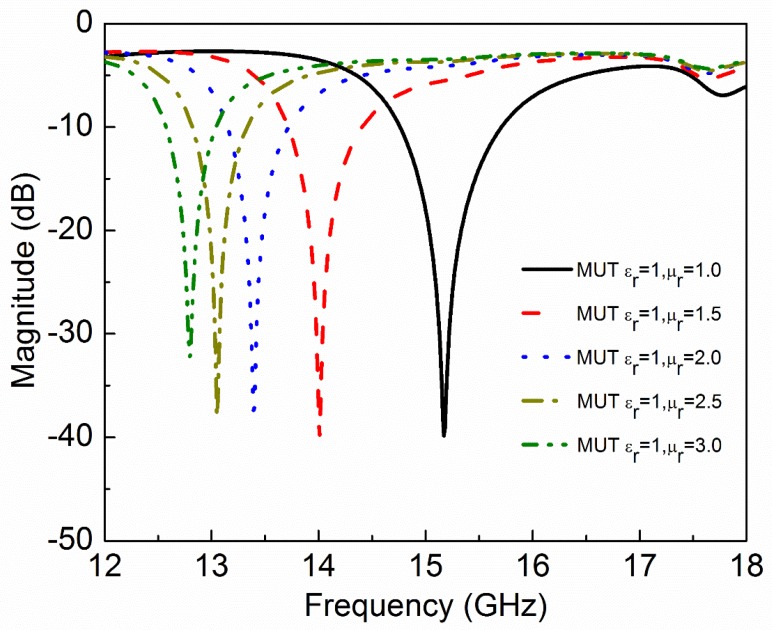
Simulated transmission coefficient (S_21_) of the sensor due to interaction with the MUT with different values of permeability (µ_r_). As the relative permeability of MUT increases, the resonance frequency decreases.

**Figure 7:**
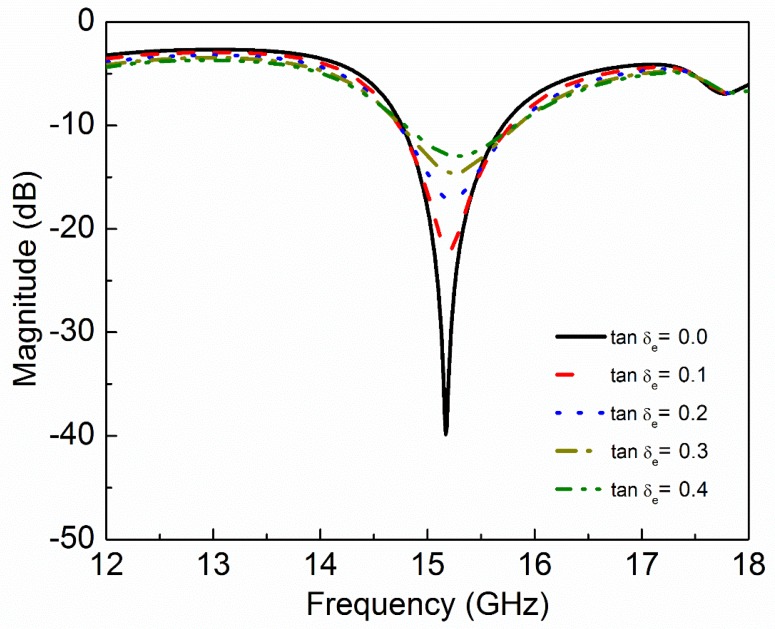
Simulated transmission coefficient (S_21_) of the sensor due to interaction with the MUT with different values of dielectric loss tangent (tanδ_e_).

**Figure 8:**
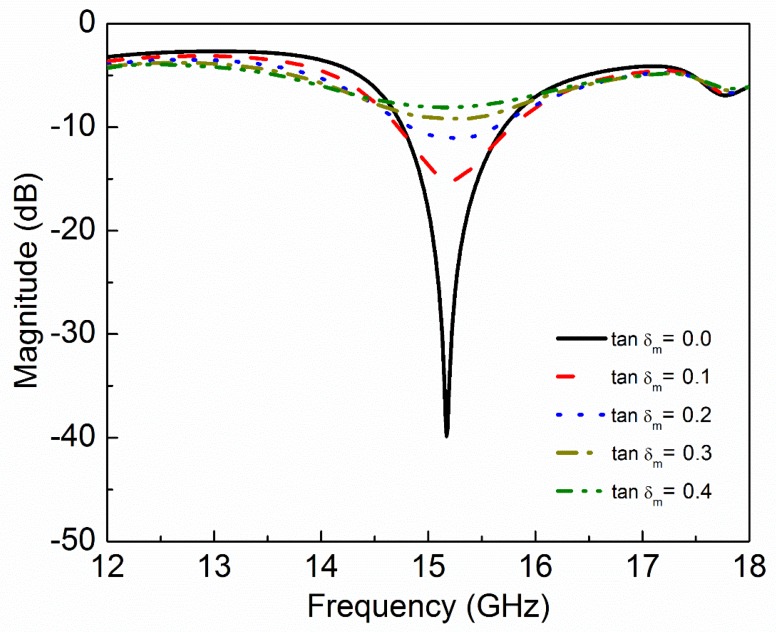
Simulated transmission coefficient (S_21_) of the sensor due to interaction with the MUT with different values of magnetic loss tangent (tanδ_m_).

**Figure 9:**
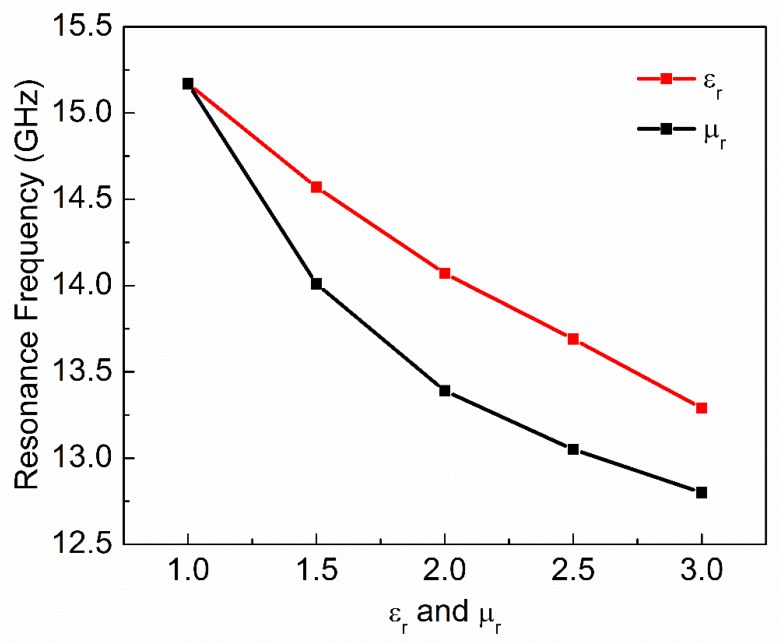
Real relative permittivity and permeability of MUT versus resonance frequency of the sensor.

**Figure 10:**
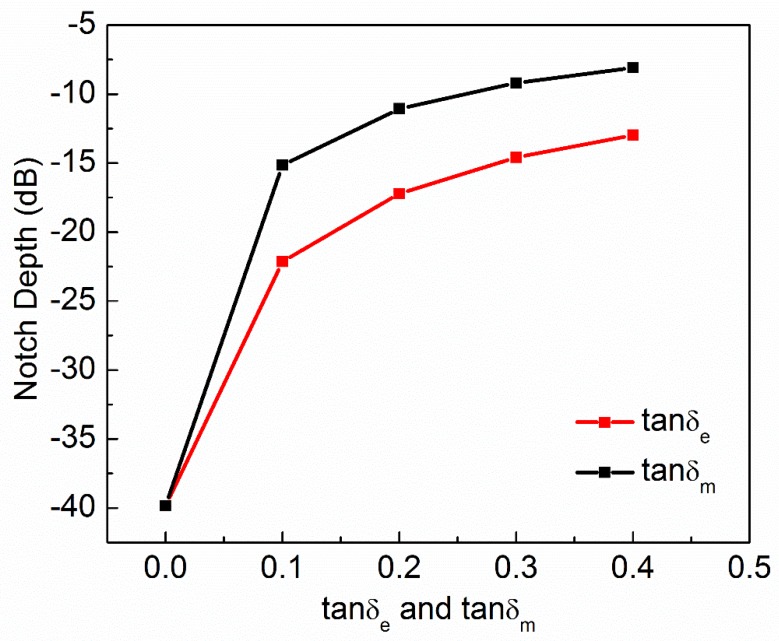
Dielectric loss tangent and magnetic loss tangent of MUT versus notch depth of the sensor.

The magnitude of the electric field elucidates the most sensitive region of the sensor, which is plotted in Figure 3. To check the sensitivity of the proposed sensor, MUT is placed at the CSSSR with constant dimensions (d_1_ = 10 mm, d_2_ = 10 mm, d_3_ = 1 mm) and an air gap of 35 µm that is equal to the thickness of the copper layer etched out in the ground plane as shown in Figure 4. For microwave materials, the complex permittivity ɛ and permeability µ can be express by the following relations [63]:

(4) $$\varepsilon =\varepsilon^{\prime }-j\varepsilon^{\prime \prime }=\varepsilon^{\prime }(1-j\tan\delta_{e})$$

(5) $$\mu =\mu^{\prime }-j\mu^{\prime \prime }=\mu^{\prime }(1-j\tan\delta_{m})$$

where $\varepsilon^{\prime }$ and $\varepsilon^{\prime \prime }$ are real and imaginary parts of complex permittivity respectively. $\mu^{\prime }$ and $\mu^{\prime \prime }$ are real and imaginary parts of complex permeability, respectively. $\tan\delta_{e}$ and $\tan\delta_{m}$ are dielectric and magnetic loss tangent, respectively. Natural materials are usually characterized by real relative permittivity $\varepsilon_{r}$ with $\varepsilon^{\prime }=\varepsilon_{r}\varepsilon_{0}$ and real relative permeability with $\mu_{r}$ with $\mu^{\prime }=\mu_{r}\mu_{0}$ at specific frequencies. Now these four parameters ($\varepsilon_{r},\mu_{r},\tan\delta_{e},\tan\delta_{m}$) of MUT are used to check the sensitivity of the proposed sensor. The other parameters like langde G factor = 2, bulk conductivity = 0 siemens/m, magnetic saturation = 0 tesla, mass density = 1.1614 kg/m^3^ are the same for all the MUTs. First, $\varepsilon_{r}$ of MUT is varied from 1 to 3 keeping other parameters constant as given in Table 2 and the effect of $\varepsilon_{r}$ on the transmission coefficient of the sensor is plotted in Figure 5. According to simulated results, as $\varepsilon_{r}$ of MUT increases from 1 to 3, the *f*_0_ decreases from 15.17 GHz to 13.29 GHz while notch depth increases from −39.84 dB to −42.32 dB. Second, $\mu_{r}$ of MUT is varied from 1 to 3 keeping other parameters constant as given in Table 3 and the effect of $\mu_{r}$ on the transmission coefficient of the sensor is plotted in Figure 6. According to simulated results, as $\mu_{r}$ of MUT increases from 1 to 3, the *f*_0_ decreases from 15.17 GHz to 12.80 GHz and notch depth also decreases from −39.84 dB to −32.32 dB. Third, $\tan\delta_{e}$ of MUT is varied from 0 to 0.4 keeping other parameters constant as given in Table 4 and the effect of $\tan\delta_{e}$ on the transmission coefficient of the sensor is plotted in Figure 7. According to simulated results, as $\tan\delta_{e}$ of MUT increases from 0 to 0.4, the *f*_0_ increases from 15.17 GHz to 15.28 GHz and notch depth decreases from −39.84 dB to −12.96 dB. Fourth, $\tan\delta_{m}$ of MUT is varied from 0 to 0.4 keeping other parameters constant as given in Table 5 and the effect of $\tan\delta_{m}$ on the transmission coefficient of the sensor is plotted in Figure 8. According to simulated results, as $\tan\delta_{m}$ of MUT increases from 0 to 0.4, the *f*_0_ increases from 15.17 GHz to 15.30 GHz and notch depth decreases from −39.84 dB to −8.08 dB. These simulated results show that $\varepsilon_{r}$ and $\mu_{r}$ affect the resonance frequency of the sensor as shown in Figure 9. While $\tan\delta_{e}$ and $\tan\delta_{m}$ affect the notch depth of the sensor as shown in Figure 10. However, all these factors do not have the same effect on resonance frequency and notch depth which means that we can distinguish the effect of each factor individually using the proposed sensor. 

## 3. Measurement and Sensitivity Analysis

**Figure 11:**
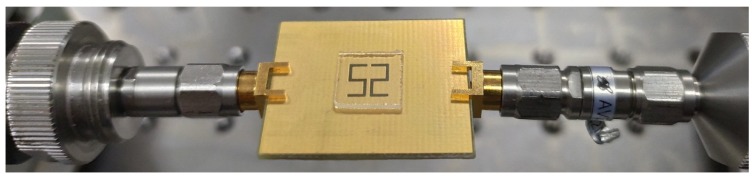
Photograph of fabricated prototype of the sensor based on CSSSR with glass as MUT and high-precision SMA connectors (impedance = 50 Ω, center contact resistance ≤0.3 mΩ, insulation resistance ≥3000 MΩ).

**Figure 12:**
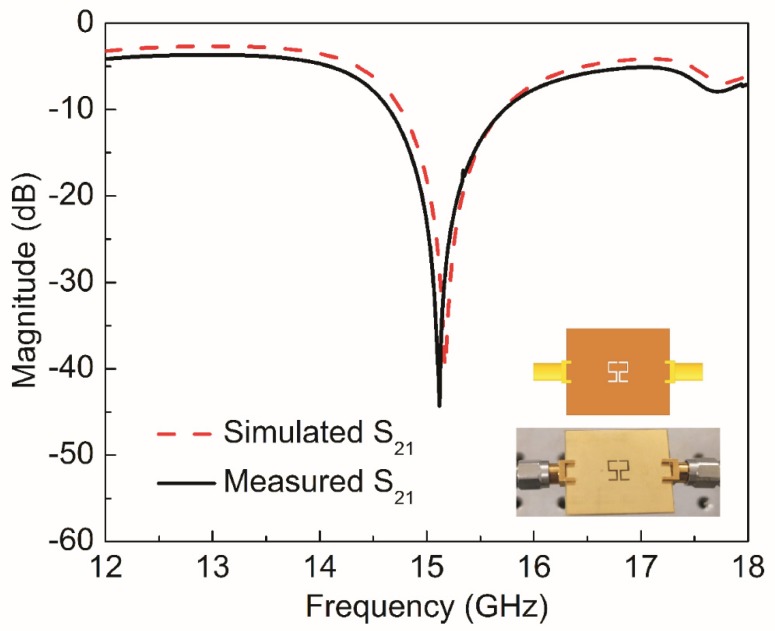
Magnitude of transmission (*S*_21_) coefficient for simulated and measured sensor based on CSSSR. Simulated resonance frequency is 15.17 GHz with notch depth of −39.84 dB while measured resonance frequency is 15.12 GHz with notch depth of −44.33 dB.

**Figure 13:**
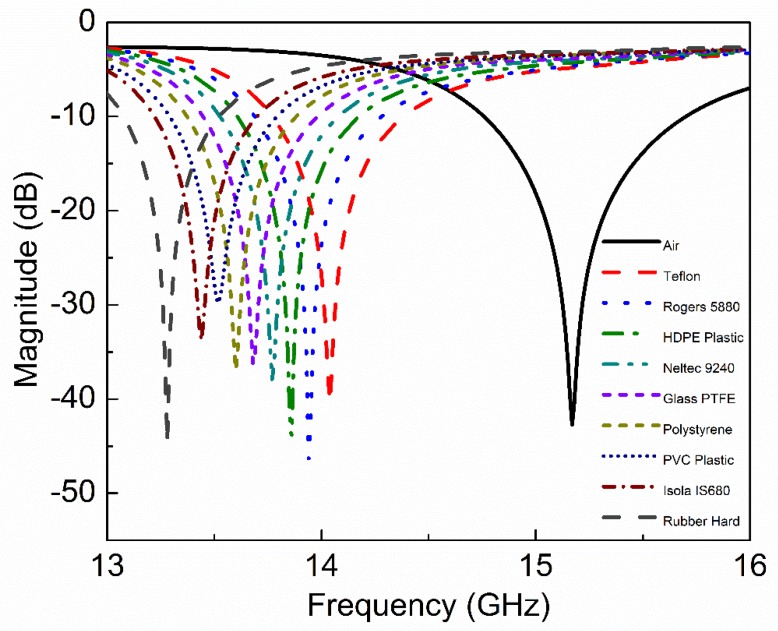
Measured transmission coefficient (S_21_) of the sensor due to interaction with composite MUTs.

The CSSSR sensor is fabricated on commercially available 1.6 mm FR4 substrate using the standard photolithographic technique which is simple and inexpensive. To connect the sensor with the vector network analyzer (VNA AV3672C), a pair of high-precision subminiature version A (SMA) connectors are connected at both side of microstrip transmission line. The impedance, center contact resistance and insulation resistance of the SMA connecters are 50 Ω, ≤ 0.3 mΩ, ≥ 3000 MΩ respectively. The frequency range, resolution and accuracy of VNA AV3672C are 10 MHz~40 GHz, 1 Hz, ± 1 × 10^-7^ respectively. The fabricated prototype of the CSSSR sensor with SMA connectors and MUT is shown in Figure 11. The VNA AV3672C is calibrated using a 2.4 mm calibration kit (AV31123) with frequency sweep 12 GHz to 18 GHz, number of sweeping points 1601 and IF-bandwidth 100Hz. After calibration the transmission coefficient of the fabricated sensor is measured and plotted in Figure 12. The measured *f*_0_ of the fabricated sensor is 15.12 GHz with notch depth of −44.33 dB and the unloaded quality factor is 501. The difference between simulated and measured resonance frequency, notch depth and quality factor is 0.05 GHz, −4.49 dB and 4, respectively, which can be attributed to fabrication tolerance. To verify the sensitivity of fabricated sensor following low permittivity materials Teflon, Rogers RT5880, high density poly ethylene (HDPE) plastic, Netltec NX9240, glass polytetrafluoroethylene (PTFEreinf), polystyrene, polyvinyl chloride (PVC) plastic, Isola IS680-280, and Rubber Hard are selected. These MUTs are placed on the CSSSR and loaded resonance frequency of the sensor is calculated. Figure 13 shows the measured S_21_ of the sensor due to interaction with composite MUTs.

**Figure 14:**
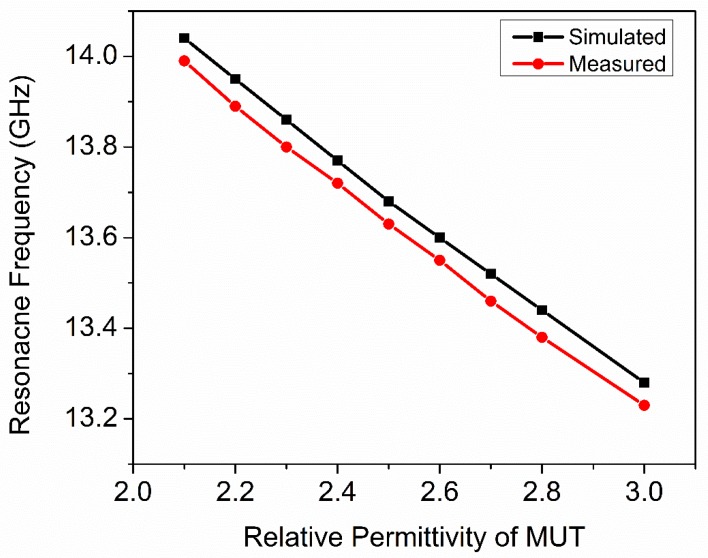
Real relative permittivity of MUT versus resonance frequency of the sensor. Relative permittivity of the MUT is inversely proportional to the resonance frequency of the sensor.

Measured *f*_0_ of the sensors due to interaction with Teflon, Rogers RT5880, HDPE plastic, Netltec NX9240, glass PTFEreinf, polystyrene, PVC plastic, Isola IS680-280, and rubber hard are 13.99 GHz, 13.89 GHz, 13.80 GHz, 13.72 GHz, 13.63 GHz, 13.55 GHz, 13.46 GHz, 13.38 GHz, and 13.23 GHz respectively. The electromagnetic properties of MUTs and loaded resonance frequency of the sensor are tabulated in Table 6. As the MUT’s real permittivity is more significant than the real permeability, therefore the real relative permittivity of MUT versus simulated and measured *f*_0_ of the sensor is plotted in Figure 14. The percentage error is 0.35% between measured and simulated resonance frequencies of the fabricated sensor when loaded with Teflon as MUT. The percentage error is 0.43% between simulated and measured resonance frequencies of the fabricated sensor when loaded with Rogers RT5880 and HDPE plastic as MUT. The percentage error is 0.36% between simulated and measured resonance frequencies of the fabricated sensor when loaded with Netltec NX9240, glass PTFEreinf and polystyrene as MUT. The percentage error is 0.44% between measured and simulated resonance frequencies of the fabricated sensor when loaded with PVC plastic and Isola IS680-280 as MUT. The percentage error is 0.37% between measured and simulated resonance frequencies of the fabricated sensor when loaded with rubber hard as MUT. The slope of Figure 14 is the sensitivity of the proposed sensor which is the ratio of differential output (*f*_d_ = *f*_u_-*f*_l_) and differential input (ɛ_rd_ = ɛ_r2_-ɛ_r1_). The differential sensitivity of composite MUTs is calculated with respect to Air (ε_r_ = 1.0006, and *f*_0_ = 15.12) and tabulated in Table 7. The following relation is used to calculate the sensitivity of the fabricated sensor, as given in [47]:

(6) $$S=\frac{\partial f_{d}}{\partial \varepsilon_{rd}}=\underset{∆\varepsilon_{r2}-∆\varepsilon_{r1}\to 0}{\lim}\frac{∆f_{u}-∆f_{l}}{∆\varepsilon_{r2}-∆\varepsilon_{r1}}$$

The relationship between the measured resonance frequencies and real relative permittivity of the MUT can be approximated by a polynomial function, as given in [64]:

(7) $$f_{r,MUT}=A_{1}+A_{2}\varepsilon_{r}+A_{3}\varepsilon_{r}{}^{2}$$

The constant values (*A*_1_, *A*_2_, *A*_3_) of the polynomial are extracted using measured resonance frequencies ($f_{r,MUT}$) of the sensor due to interaction with the MUT with different values of real relative permittivity ($\varepsilon_{r}$). Finally, the transcendental equation becomes:

(8) $$f_{r,MUT}=16.462-1.411\varepsilon_{r}+0.112\varepsilon_{r}{}^{2}$$

Equation (8) can predict the resonance frequency of the sensor due to interaction with an MUT with real relative permittivity ranges from 2.1 to 3. Equation (8) can be rearranged to calculate relative permittivity in the following form:

(9) $${\varepsilon^{\prime }}_{r}=\frac{1.411-\sqrt{1.99-0.448\left(16.462-f_{r,MUT}\right)}}{0.224}$$

Equation (9) can be used to calculate the relative permittivity of an unknown MUT by measuring the resonance frequency of the sensor due to interaction with the unknown MUT. The relative sensitivity is most important parameter to compare the performance of with other sensors. The relative sensitivity of the microwave sensor based on permittivity perturbation is defined as [65]:

(10) $$S_{\varepsilon_{r}}=\frac{f_{r,MUT}-f_{0}}{f_{0}\left(\varepsilon_{r}-1\right)}\times 100$$

where f_r,MUT_ is the resonance frequency of the sensor due to interaction with the MUT with relative permittivity ε_r_. The relative sensitivity of various state of the art sensors [66,67,68,69,70] is tabulated in Table 8.

## 4. Conclusions

This paper presents an extremely sensitive microwave sensor that is based on a microstrip transmission line and complementary symmetric S shape resonator (CSSSR) to measure the relative permittivity of low permittivity materials. The proposed sensor is sensitive for the four parameters of MUT ($\varepsilon_{r},\mu_{r},\tan\delta_{e},\tan\delta_{m}$) which is demonstrated by electromagnetic simulation. It is concluded that the fringing electromagnetic fields of CSSSR interact with the MUT, and real relative permittivity and permeability which are greater than unity cause a decrease in the resonance frequency while dielectric and magnetic loss tangents cause a decrease in the magnitude of the transmission coefficient. After fabrication, the fabricated sensor is used to measure composite MUTs and relative sensitivity of 6.7% is achieved with a measurement error less than 0.5%. The transcendental equation for the CSSSR sensor is derived by approximating the resonance frequencies of the sensor with respect to the relative permittivity of composite MUTs.

## Figures and Tables

**Table 1:** Comparison of frequency shift with various state of art sensors.

Ref	Hosting Transmission Line	Resonating Structure	Frequency Band (GHz)	Shift in Resonance Frequency
[49]	Microstrip	Rectangular CSRR	0.8–1.3	38%
[50]	Microstrip	Rectangular CSRR	1.8–2.8	36%
[51]	Microstrip	Single Compound CSRR	1.08–1.63	34%
[52]	Microstrip	Circular CSRR	1.75–2.75	36%
[53]	Microstrip	Hexagonal CSRR	5.23–8.45	38%
[54]	Microstrip	Single CSRR	0.90–10.90	50%
This Work	Microstrip	CSSSR	13.28–15.17	100%

**Table 2:** Effect of relative permittivity of MUT on resonance frequency and notch depth of the sensor.

Material Under Test (MUT)				Resonance Frequency	Notch Depth
ε_r_	µ_r_	tanδ_e_	tanδ_m_	GHz	dB
1.0	1	0	0	15.17	−39.84
1.5	1	0	0	14.57	−44.88
2.0	1	0	0	14.07	−38.41
2.5	1	0	0	13.69	−40.77
3.0	1	0	0	13.29	−42.32

**Table 3:** Effect of relative permeability of MUT on resonance frequency and notch depth of the sensor.

Material Under Test (MUT)				Resonance Frequency	Notch Depth
ε_r_	µ_r_	tanδ_e_	tanδ_m_	GHz	dB
1	1.0	0	0	15.17	−39.84
1	1.5	0	0	14.01	−40.11
1	2.0	0	0	13.39	−37.41
1	2.5	0	0	13.05	−37.93
1	3.0	0	0	12.80	−32.23

**Table 4:** Effect of dielectric loss tangent of MUT on resonance frequency and notch depth of the sensor.

Material Under Test (MUT)				Resonance Frequency	Notch Depth
ε_r_	µ_r_	tanδ_e_	tanδ_m_	GHz	dB
1	1	0.0	0	15.17	−39.84
1	1	0.1	0	15.20	−22.15
1	1	0.2	0	15.22	−17.21
1	1	0.3	0	15.25	−14.60
1	1	0.4	0	15.28	−12.96

**Table 5:** Effect of magnetic loss tangent of MUT on resonance frequency and notch depth of the sensor.

Material Under Test (MUT)				Resonance Frequency	Notch Depth
ε_r_	µ_r_	tanδ_e_	tanδ_m_	GHz	dB
1	1	0	0.0	15.17	−39.84
1	1	0	0.1	15.21	−15.15
1	1	0	0.2	15.28	−11.06
1	1	0	0.3	15.32	−9.19
1	1	0	0.4	15.30	−8.08

**Table 6:** Simulated and measured resonance frequencies of the sensor due to interaction with MUTs.

Material Under Test (MUT)	MUT Properties				Simulated Resonance Frequency	Measured Resonance Frequency
	ε_r_	µ_r_	tanδ_e_	tanδ_m_	GHz	GHz
Teflon(tm)	2.1	1	0.001	0	14.04	13.99
Rogers RT5880	2.2	1	0.0009	0	13.95	13.89
HDPE Plastic	2.3	1	0.0005	0	13.86	13.80
Neltec NX9240	2.4	1	0.0016		13.77	13.72
Glass PTFEreinf	2.5	1	0.002	0	13.68	13.63
Polystyrene	2.6	1	0	0	13.60	13.55
PVC Plastic	2.7	1	0.007	0	13.52	13.46
Isola IS680-280	2.8	1	0.0025	0	13.44	13.38
Rubber Hard	3	1	0	0	13.28	13.23

**Table 7:** Differential sensitivity of the fabricated sensor with respect to MUT.

Material Under Test (MUT)	Differential Input	Differential Output	Differential Sensitivity
	$\varepsilon_{MUT}-\varepsilon_{Air}$	$f_{u}-f_{l}$	**%**
Teflon(tm)	1.0994	1.13	102
Rogers RT5880	1.1994	1.23	102
HDPE Plastic	1.2994	1.32	101
Netltec NX9240	1.3994	1.40	100
Glass PTFEreinf	1.4994	1.49	99
Polystyrene	1.5994	1.57	98
PVC Plastic	1.6994	1.66	98
Isola IS680-280	1.7994	1.74	97
Rubber Hard	1.9994	1.89	95

**Table 8:** Comparison of relative sensitivity with various state of the art sensors.

Ref	Resonating Structure	Resonance Frequency (GHz)	Permittivity Range Studied	Relative Sensitivity (%)
[65]	SIR	1.91	10–80	0.84
[66]	CSRR	2.4	10–80	0.61
[67]	SRR	1.72	10–80	0.78
[68]	Open-Loop Resonators	2.6	10–140	1.49
[69]	SRR	1.8	2.42–22.52	3.04
[70]	CSRR	2.5	2.93–3.64	3.58
This Work	CSSSR	15.12	2.1–3	6.7
